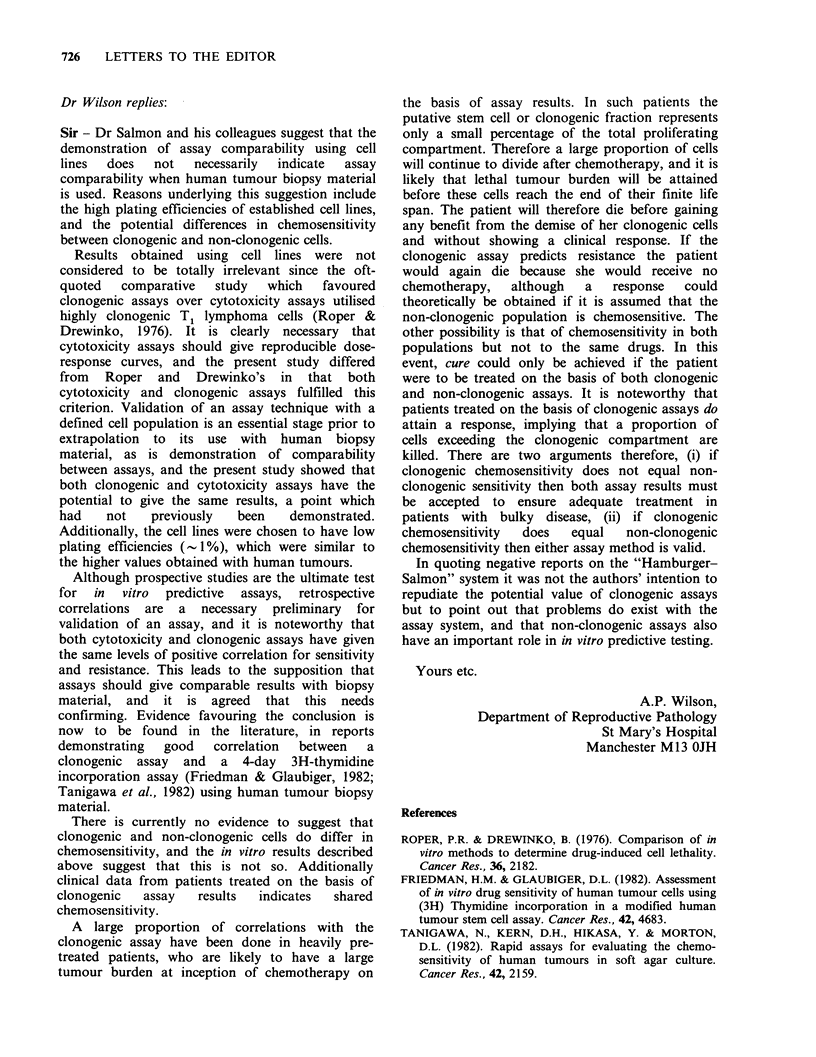# Comparison of assays used for in vitro chemosensitivity testing of human tumours

**Published:** 1984-11

**Authors:** A.P. Wilson


*Dr Wilson replies*:

**Sir** – Dr Salmon and his colleagues suggest that the demonstration of assay comparability using cell lines does not necessarily indicate assay comparability when human tumour biopsy material is used. Reasons underlying this suggestion include the high plating efficiencies of established cell lines, and the potential differences in chemosensitivity between clonogenic and non-clonogenic cells.

Results obtained using cell lines were not considered to be totally irrelevant since the oft-quoted comparative study which favoured clonogenic assays over cytotoxicity assays utilised highly clonogenic $T_1$ lymphoma cells (Roper & Drewinko, 1976). It is clearly necessary that cytotoxicity assays should give reproducible dose-response curves, and the present study differed from Roper and Drewinko's in that both cytotoxicity and clonogenic assays fulfilled this criterion. Validation of an assay technique with a defined cell population is an essential stage prior to extrapolation to its use with human biopsy material, as is demonstration of comparability between assays, and the present study showed that both clonogenic and cytotoxicity assays have the potential to give the same results, a point which had not previously been demonstrated. Additionally, the cell lines were chosen to have low plating efficiencies ($\sim 1\%$), which were similar to the higher values obtained with human tumours.

Although prospective studies are the ultimate test for *in vitro* predictive assays, retrospective correlations are a necessary preliminary for validation of an assay, and it is noteworthy that both cytotoxicity and clonogenic assays have given the same levels of positive correlation for sensitivity and resistance. This leads to the supposition that assays should give comparable results with biopsy material, and it is agreed that this needs confirming. Evidence favouring the conclusion is now to be found in the literature, in reports demonstrating good correlation between a clonogenic assay and a 4-day 3H-thymidine incorporation assay (Friedman & Glaubiger, 1982; Tanigawa *et al.*, 1982) using human tumour biopsy material.

There is currently no evidence to suggest that clonogenic and non-clonogenic cells do differ in chemosensitivity, and the *in vitro* results described above suggest that this is not so. Additionally clinical data from patients treated on the basis of clonogenic assay results indicates shared chemosensitivity.

A large proportion of correlations with the clonogenic assay have been done in heavily pre-treated patients, who are likely to have a large tumour burden at inception of chemotherapy on the basis of assay results. In such patients the putative stem cell or clonogenic fraction represents only a small percentage of the total proliferating compartment. Therefore a large proportion of cells will continue to divide after chemotherapy, and it is likely that lethal tumour burden will be attained before these cells reach the end of their finite life span. The patient will therefore die before gaining any benefit from the demise of her clonogenic cells and without showing a clinical response. If the clonogenic assay predicts resistance the patient would again die because she would receive no chemotherapy, although a response could theoretically be obtained if it is assumed that the non-clonogenic population is chemosensitive. The other possibility is that of chemosensitivity in both populations but not to the same drugs. In this event, *cure* could only be achieved if the patient were to be treated on the basis of both clonogenic and non-clonogenic assays. It is noteworthy that patients treated on the basis of clonogenic assays *do* attain a response, implying that a proportion of cells exceeding the clonogenic compartment are killed. There are two arguments therefore, (i) if clonogenic chemosensitivity does not equal non-clonogenic sensitivity then both assay results must be accepted to ensure adequate treatment in patients with bulky disease, (ii) if clonogenic chemosensitivity does equal non-clonogenic chemosensitivity then either assay method is valid.

In quoting negative reports on the "Hamburger–Salmon" system it was not the authors' intention to repudiate the potential value of clonogenic assays but to point out that problems do exist with the assay system, and that non-clonogenic assays also have an important role in *in vitro* predictive testing.

Yours etc.

A.P. Wilson,
Department of Reproductive Pathology
St Mary's Hospital
Manchester M13 0JH

**References**

ROPER, P.R. & DREWINKO, B. (1976). Comparison of *in vitro* methods to determine drug-induced cell lethality. *Cancer Res.*, **36**, 2182.

FRIEDMAN, H.M. & GLAUBIGER, D.L. (1982). Assessment of *in vitro* drug sensitivity of human tumour cells using (3H) Thymidine incorporation in a modified human tumour stem cell assay. *Cancer Res.*, **42**, 4683.

TANIGAWA, N., KERN, D.H., HIKASA, Y. & MORTON, D.L. (1982). Rapid assays for evaluating the chemo-sensitivity of human tumours in soft agar culture. *Cancer Res.*, **42**, 2159.